# Morphology and Molecular Features of Rare Colorectal Carcinoma Histotypes

**DOI:** 10.3390/cancers11071036

**Published:** 2019-07-23

**Authors:** Andrea Remo, Matteo Fassan, Alessandro Vanoli, Luca Reggiani Bonetti, Valeria Barresi, Fabiana Tatangelo, Roberta Gafà, Guido Giordano, Massimo Pancione, Federica Grillo, Luca Mastracci

**Affiliations:** 1 Pathology Unit, Services Department, ULSS9 “Scaligera”, 37122 Verona, Italy; 2 Department of Medicine (DIMED), Surgical Pathology Unit, University of Padua, 35100 Padua, Italy; 3 Unit of Anatomic Pathology, Department of Molecular Medicine, University of Pavia and Fondazione IRCCS Policlinico San Matteo, 27100 Pavia, Italy; 4 Department of Diagnostic, Clinic and Public Health Medicine, Anatomic Pathology, University of Modena and Reggio Emilia, 41121 Modena, Italy; 5 Department of Diagnostic and Public Health, Section of Pathology, University of Verona, 37134 Verona, Italy; 6 Department of Pathology, Istituto Nazionale Tumori Fondazione G. Pascale, IRCCS, 80131 Naples, Italy; 7 Section of Anatomic Pathology, Department of Morphology, Surgery and Experimental Medicine, University of Ferrara and S. Anna University Hospital, 44121 Ferrara, Italy; 8 U.O.C. Oncologia Medica, Ospedali Riuniti Azienda Ospedaliera Universitaria, 71122 Foggia, Italy; 9 Department of Sciences and Technologies, University of Sannio, 82100 Benevento, Italy; 10 Anatomic Pathology, Department of Integrated Surgical and Diagnostic Sciences (DISC), University of Genoa and Ospedale Policlinico San Martino, 16132 Genoa, Italy

**Keywords:** colorectal cancer histotypes, signet ring cell carcinoma, medullary carcinoma, lymphoepitelioma-like carcinoma, cribriform/comedo-type carcinoma, micropapillary carcinoma, clear cell carcinoma, hepatoid carcinoma, adenocarcinoma with osseous metaplasia, rhabdoid carcinoma

## Abstract

Several histopathological variants of colorectal carcinoma can be distinguished, some associated with specific molecular profiles. However, in routine practice, ninety/ninety-five percent of all large bowel tumors are diagnosed as conventional adenocarcinoma, even though they are a heterogeneous group including rare histotypes, which are often under-recognized. Indeed, colorectal cancer exhibits differences in incidence, location of tumor, pathogenesis, molecular pathways and outcome depending on histotype. The aim is therefore to review the morphological and molecular features of these rare variants of intestinal carcinomas which may hold the key to differences in prognosis and treatment.

## 1. Introduction

Colorectal cancer (CRC) is the third most frequent malignant neoplasm worldwide. The CRC risk increases with age, as the majority of cases are diagnosed in patients with more than 50 years of age. CRCs exhibit biological differences in both pathogenesis and molecular pathways reflecting different incidences, sidedness and outcome [1,2]. Most CRC are located in the sigmoid colon/rectum, but the proportion of carcinomas in the right colon increases with age [3]. 

Three kinds of alterations are involved in CRC development: (1) chromosomal instability (CIN); (2) microsatellite instability (MSI); (3) CpG island methylator phenotype (CIMP). A different association between these pathogenetic alterations determines distinct molecular pathways: (i) traditional, (ii) alternative and (iii) serrated [1]:

(i) The *traditional* pathway is based on APC and KRAS mutations (by CIN alterations). These neoplasms usually involve the left colon.
(ii) The *alternative* pathway is characterized by a CIMP-low phenotype, predominant KRAS and occasional BRAF mutations, with no CIN. The prognosis of these CRC is aggressive.
(iii) The *serrated* pathway is characterized by BRAF mutations and epigenomic instability (CIMP-high). These lesions are located mainly in the right colon with MSI morphology (mucinous, medullary and tumours with intraepithelial lymphocytes) or MSS with a serrated morphology (eosinophilic cytoplasm, epithelial serration and tufts and vescicular basal nuclei) [1,3].

Tumor development through the *traditional* pathway is relatively slow (5–20 years), probably due to the fact that the initial events occur in the fully differentiated cells of the colonic crypt. APC mutations, generally, are detected in the cells of the upper crypt compartment according to the top-down morphogenetic model [4]. The causal events underlying the *serrated* pathway, however, may take place in the cells of the lower crypt compartment which are less differentiated and rapidly progressive [5]. This could explain the morphologic heterogeneity of tumors arising in the right colon, in older patients which are usually BRAF mutated. These CRCs are thought to develop rapidly and may in part explain interval cancers [6]. These new insights into the molecular pathogenesis of CRC have contributed to the distinction of right- and left-sided CRCs, identifying them as two distinct clinical, pathological and molecular entities [7]. This distinction is especially useful considering the impact which sidedness may have on treatment choice.

**Figure 1:**
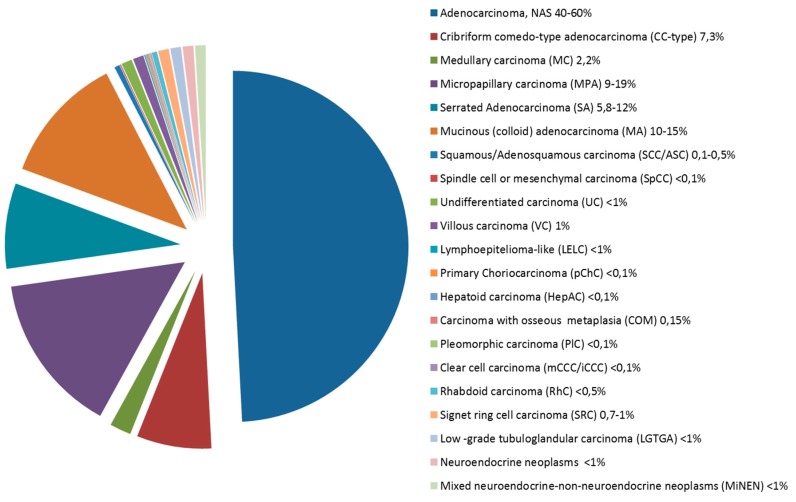
Pie chart showing the frequency of colorectal carcinomas by histologic type.

Several epithelial histopathological variants of CRC can be distinguished, some associated with specific molecular profiles. In routine practice, 90–95% of all large bowel tumors are diagnosed as classic adenocarcinoma, however this group is actually a heterogeneous population including rare histotypes which are often underdiagnosed but which may collectively reach up to 50% of CRCs in histologically classified series (Figure 1). Indeed, a frequent downfall in large studies is that CRCs are collected regardless of histotype with no importance being given to rare histotypes. The aim is to review the morphologic and molecular features of these rare histotypes which may be seen as pure/prevalent forms (Table 1) or as composite/mixed morphologically heterogeneous neoplasms (in which each separate morphological entity should be reported and quantified). The understanding of the morphological complexity of a group of tumors which are often all placed together in the CRC basket is especially important for non-pathology clinical colleagues or researchers who may not be aware or appreciate the subtleties of morphology (phenotype) with its possible molecular implications (genotype).

## 2. Serrated Adenocarcinoma (SA)

### 2.1. Background

Following the first description of five CRCs histologically resembling serrated polyps by Jass and Smith [8], Mäkinen et al. reported twenty-seven CRCs associated with an adjacent serrated adenoma [9]. They noted that such cases exhibited distinctive clinical, histological and molecular features, suggesting that serrated adenocarcinomas (SAs) may be considered a distinct entity, probably representing an end-point of the serrated pathway. Several subsequent studies have confirmed the clinico-pathologic and molecular differences between SA and conventional CRC [10,11], recognizing SA as a distinct CRC subtype in the 2010 WHO classification. SA can be identified either by the presence of a residual serrated polyp or by its peculiar histologic characteristics, even when precursor lesions are no longer visible [10,12]. Considering this definition, SA accounts for 5.8–12% of all CRCs and up to 17% of proximal CRCs. 

### 2.2. Clinical Presentation

The mean age of patients with SA ranges between 65 and 70 years; gender prevalence is controversial, with a prevalence of females in Finnish studies [8,11,12] and of males in Spanish series [10]. 

### 2.3. Sidedness

Most SA are located in either the right colon (47–57%) or the rectum (15–29%).

### 2.4. Morphologic Diagnostic Criteria

**Figure 2:**
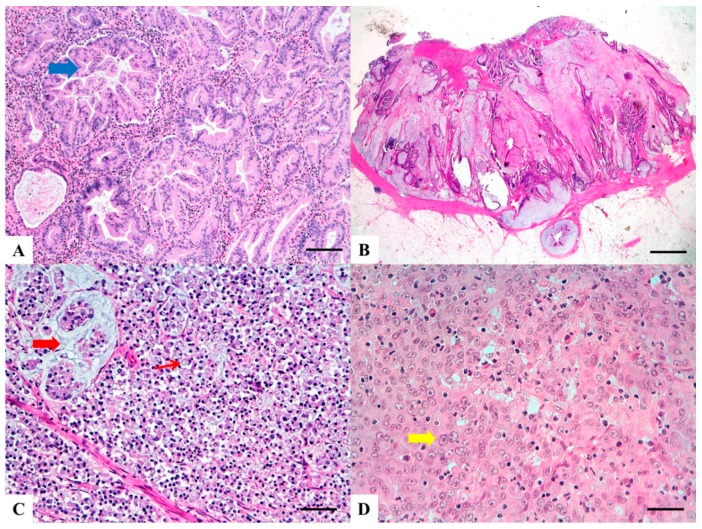
Haematoxylin and Eosin stained sections of rare type colorectal carcinomas. (**A**) Serrated Adenocarcinoma: epithelial serrations or tufts (thick blue arrow), abundant eosinophilic or clear cytoplasm, vesicular basal nuclei with preserved polarity. Scale bar 200 micron. (**B**) Mucinous Carcinoma: presence of extracellular mucin (>50%) associated with ribbons or tubular structures of neoplastic epithelium. Scale bar 2 mm. (**C**) Signet Ring Carcinoma: more than 50% of signet cells with infiltrative growth pattern (thin red arrow) or floating in large pools of mucin (thick red arrow). Scale bar 200 micron. (**D**) Medullary carcinoma: neoplastic cells with syncytial appearance (thick yellow arrow) and eosinophilic cytoplasm associated with abundant peritumoral and intratumoral lymphocytes. Scale bar 100 micron.

The histologic criteria for SA diagnosis include: epithelial serrations and tufts, abundant eosinophilic or clear cytoplasm, vesicular basal nuclei with chromatin condensation around the nuclear envelope, easily distinguishable nuclei and preserved polarity, absence or less than 10% necrosis of the total surface area and, within mucinous areas, the presence of cell balls and papillary rods [12]. Importantly, serrations of SAs are composed of epithelium with or without basement membrane, but lack the fibro-vascular cores seen in non-serrated CRCs (Figure 2A). Three growth patterns have been described-serrated, mucinous and trabecular, the latter being characteristic of poorly differentiated SAs, which may be challenging to recognize. 

### 2.5. Molecular Alterations

Gene expression and methylome profiling analysis has highlighted a clear distinction between SAs and conventional CRCs [13,14,15], showing higher representation of morphogenesis-, hypoxia-, cytoskeleton- and vesicle transport-related functions in SA. Among the most relevant differentially expressed genes, hypoxia-inducible factor 1-alpha (HIF-1α), fascin 1, the anti-apoptotic gene hippocalcin and annexin A10, are specifically upregulated in SA and, therefore, have been proposed as immunohistochemical markers for SA diagnosis. KRAS and BRAF mutations are common in SA, being found in 33% and 45% of cases, respectively, and may contribute to stabilize HIF-1α [11]. BRAF mutation, in particular, is strongly associated with serrated morphology in CRCs. Up to 20% of SA harbor microsatellite instability (MSI).

### 2.6. Prognosis

Garcia-Solano et al. [10] demonstrated that SA patients more frequently presented lymph node metastases (52%) and they showed a less favorable prognosis compared to conventional CRCs, especially in the case of left-side SA. In addition, SAs are characterized by higher frequency of adverse histologic features at the invasive front, such as high-grade tumor budding and weak peritumoural lymphocytic infiltration [16]. Interestingly, Zhu et al. highlighted that high PD-L1 expression by SAs is frequent (25%) and associated with poor survival [17]. 

## 3. Mucinous (Colloid) Adenocarcinoma (MA)

### 3.1. Background

Primary mucinous (colloid) colorectal adenocarcinoma (MA), is defined by the presence of more than 50% of extracellular mucin component containing malignant epithelium, according to the WHO 2010 classification criteria [18]. It constitutes approximately 10–15% of all CRCs and it is associated with peculiar clinico-pathological and prognostic features if compared to conventional CRCs [19].

### 3.2. Clinical Presentation

The mean age of presentation is 60 years (range from 10 to 93 years) with low female gender prevalence. Symptoms do not differ from those of conventional CRC however MA is often diagnosed in advanced stage, it is generally of larger size and it is associated with frequent loco-regional lymph node involvement and peritoneal implants [20].

### 3.3. Sidedness

MA is more often localized to the right colon [21], including caecum, ascending colon and proximal transverse colon.

### 3.4. Morphologic Diagnostic Criteria

Histology shows abundant extracellular mucin associated with ribbons or tubular structures of neoplastic epithelium (Figure 2B). Single cells, including signet ring cells, may be found floating within the mucin or attached to the adjacent stromal wall. Mucinous morphological changes in post neoadjuvant treatment cancers must not be interpreted as MA [22]. Presence of tumor infiltrating lymphocytes (TILs) is frequent, may show a Crohn-like appearance and is frequently associated with MSI [21].

### 3.5. Molecular Genotype

MA is one of the histotypes associated with MSI [20], both in sporadic and in Lynch syndrome associated CRCs. In sporadic CRCs, MSI is due to epigenetic silencing of the promoter region of mismatch repair (MMR) genes (predominantly MLH1) by CpG island hypermethylation. Diversely, Lynch syndrome associated patients show germline mutational inactivation of genes encoding the MMR proteins MLH1, MSH2, MSH6 or PMS2 and MA has a 22–40% prevalence in this setting. Furthermore the high mutational load of MA MSI-H, with its high production of tumor specific neoantigens, represents a strong immunogenic factor which explains the presence of TILs. MA has a greater incidence (65%) of KRAS mutations, compared to other CRC sub-types without mucin production, and often shows BRAF mutation.

### 3.6. Prognosis

There is no definitive evidence regarding prognostic differences between MA compared to conventional CRC. Indeed, the Literature is a well of conflicting data about prognosis and overall survival; in this context sidedness may have an impact (poorer prognosis in rectal MA versus colonic MA) [21]. A recent study has shown that there is no difference in prognosis, adjusted for stage, between non mucinous conventional adenocarcinoma and adenocarcinomas with mucin production of any percentage [20] making the 50% cut off debatable.

## 4. Signet Ring Cell Carcinoma (SRC)

### 4.1. Background 

Primary colorectal signet ring cell carcinoma (SRC) is a rare histotype representing about 0.7–1% of all CRCs. It was first described by Laufman and Saphir in 1951 [23] and is defined as a CRC variant with > 50% of tumour cells showing prominent intracytoplasmic mucin [18]. 

### 4.2. Clinical Presentation

This subtype of CRC occurs in younger individuals compared to conventional CRC (range at diagnosis 48 to 70 years) and it seems to occur more frequently in females.

### 4.3. Sidedness

SRC has been reported to be more frequently localized in the right colon, including caecum, ascending colon and proximal transverse colon even though discrepancies regarding site of tumor are present in the literature [24].

### 4.4. Morphologic Diagnostic Criteria

SRC is characterized by more than 50% of cells with prominent intracytoplasmic mucin and displacement of the nucleus (Figure 2C) [18]. These cells can be found associated with two main histological patterns of growth: (a) infiltrative, *mucin poor* (linitis plastica-like) pattern, often associated with adverse histological features such as vascular and perineural invasion; (b) *mucin rich* pattern with signet ring cells floating in large pools of mucin. There is no standardized method to separate SRC into the two morphological groups [25]. Adenocarcinomas with presence of less than 50 % of signet ring cells are defined as “adenocarcinomas with signet ring cell component” [18]. As for MA, high intra and peritumoral TILs are seen, especially in MSI associated SRC.

### 4.5. Molecular Genotype

SRC share molecular features with MA: they have a higher frequency of KRAS and BRAF mutations compared with conventional CRC, which are associated with a shorter median OS compared to KRAS and BRAF wild-type patients. SRC are often MSI-H tumors and have CpG island methylator phenotype-high (CIMP-H). Though SRC are a clinically aggressive tumours, MSI-H status correlates with a better prognosis and should be considered low grade tumors [26]. Two different molecular genotypes in SRC have been recognized: (a) the *hypermethylated genotype* with MSI-H, CIMP-H, BRAF-*V600E*, PDL1+, predominantly located in the right colon, which may be treated with immune checkpoint inhibitor therapy; (b) the *hypomethylated genotype*, predominantly located in left colon [27]. 

### 4.6. Prognosis

SRC is diagnosed at a more advanced stage with transmural extension, loco-regional lymph node metastases and peritoneal dissemination [28]. SRC has been considered an extremely aggressive tumor and is considered as an independent histologic prognostic factor of less favorable outcome and high risk of death [28]. However, as stated above, due to its heterogeneity and different molecular genotypes, SRC should not always be considered predictor of poor prognosis: SRC MMR/MSI-H should be considered as low grade tumors whereas SRC MSS/MSI low show aggressive behavior even though these data are still being debated. Another feature of promising prognostic value is represented by mucin phenotype. Some literature data have demonstrated that mucin-poor SRC have a worse prognosis, with an aggressive clinical outcome if compared with mucin-rich SRC [25].

## 5. Medullary Carcinoma (MC)

### 5.1. Background

The term “medullary (adeno)carcinoma” (MC) of the colon was first employed by Jessurun et al. in 1999 [29]. Subsequently, small series of MCs were reported and described as a distinct subgroup of CRCs showing minimal glandular differentiation and intense intratumoral and peritumoral lymphocytic infiltration [29,30,31]. These tumors were also characterized by proximal location, diploid flow cytometric nuclear DNA content and low levels of p53 protein expression. In addition, they demonstrated near always MSI by molecular analysis and better survival rate than other poorly differentiated CRCs [29,30,31]. Further studies confirmed and expanded these initial observations [32].

### 5.2. Clinical Presentation

The mean age of patients with MC is similar to that of patients with conventional CRC, with a prevalence in the female gender. MCs account for a small percentage (2.2%) of all CRCs, but represents about 20% of large bowel poorly differentiated adenocarcinomas [33]. MCs may be sporadic or develop in patients with Lynch syndrome.

### 5.3. Sidedness

The large majority of MCs are located in the proximal colon [32,33].

### 5.4. Morphologic Diagnostic Criteria

MC is characterized by neoplastic cells with vesicular nuclei, prominent nucleoli and abundant eosinophilic cytoplasm, arranged in solid sheets and exhibiting prominent infiltration by intraepithelial lymphocytes (Figure 2D). The percentage of the tumor area which should exhibit medullary features in order to classify a tumor as medullary is not specified. In a recent meta-analysis, Pyo and coworkers [33] reported a great variation in the histologic criteria utilized for the definition of MC among different studies. In addition some Authors considered presence of MMR deficiency necessary for the diagnosis of MC [31,32]. For these reasons, the histological diagnosis of MC results poorly reproducible and the diagnostic criteria for differentiating MCs from non-medullary poorly differentiated carcinomas still need to be clarified [34].

### 5.5. Molecular Genotype

Most MCs are MSI-H at molecular analysis and show loss of expression of MMR proteins (generally MLH1 and PMS2) by immunohistochemistry. Moreover, MCs often demonstrate MLH1 promoter methylation and BRAF-*V600E* mutation. On the contrary, TP53 and KRAS mutations occur much less frequently in MC than in conventional CRC [33]. Immunophenotypically, MCs often show loss of CDX2 and cytokeratin (CK) 20 expression and positivity for calretinin [35,36]. The clinicopathologic and molecular features of MC are mainly related to their MMR deficient phenotype. However, recent studies indicate that MCs differ from the other types of MSI-H CRCs especially regarding the tumor immunoregulatory microenvironment [37,38].

### 5.6. Prognosis

MCs have a more favorable clinical outcome as compared to conventional poorly differentiated adenocarcinomas but not with respect to CRCs when considered globally [33]. Recent studies suggest that MCs seem to behave more aggressively than other MSI-H CRCs [39].

## 6. Lymphoepitelioma-Like Carcinoma (LELC)

### 6.1. Background

Lymphoepitelioma-like carcinoma (LELC) is an undifferentiated carcinoma with prominent lymphoid stroma, found most frequently in the nasopharynx and associated with Epstein Bar Virus (EBV)-infection, although various other sites have been described [40]. In the gastrointestinal tract, the stomach is the most frequent site, while only 9 case have been described in the colon-rectum [41].

### 6.2. Clinical Presentation

Patient’s age is variable (range 25–86 years) with no gender predilection. Clinical presentation is unremarkable [40].

### 6.3. Sidedness

LELCs are present in the whole colon-rectum with the most frequent site being the sigmoid colon. 

### 6.4. Morphologic Diagnostic Criteria

**Figure 3:**
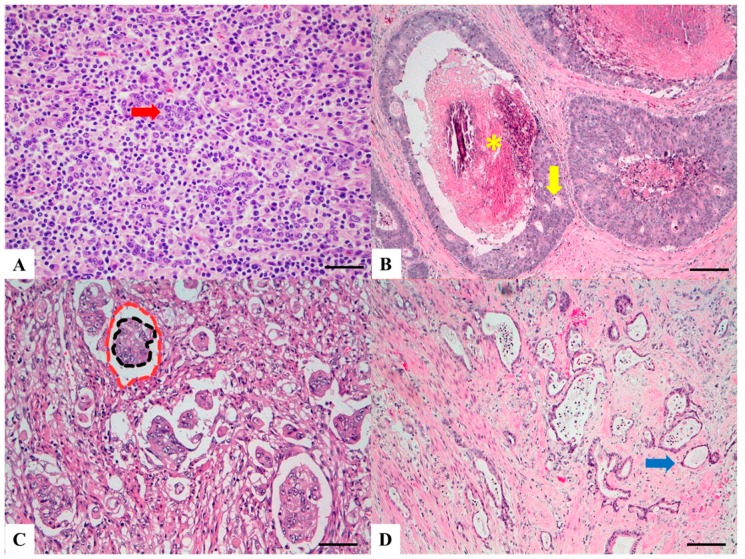
Haematoxylin and Eosin stained sections of rare type colorectal carcinomas. (**A**) Lymphoepitelioma-like carcinoma: poorly differentiated cells (red arrow) arranged in solid nests, tubules and trabeculae with poorly demarcated, infiltrative margins; intratumoral lymphoid infiltrate is extremely abundant. Scale bar 200 micron. (**B**) Cribiform comedo-type carcinoma: cribriform gland (yellow arrow) with central necrosis comedo-like (yellow asterisk). Scale bar 400 micron. (**C**) Micropapillary Carcinoma: small, tight round to oval cohesive clusters of neoplastic cells (>5 cells) floating in clear spaces (double circle red-black), without endothelial lining and with no evidence of inflammatory cells. Scale bar 200 micron. (**D**) Low grade tubulo-glandular carcinoma: very well-differentiated invasive glands with uniform circular or tubular profiles (blue arrow) with bland cytologic atypia. Scale bar 400 micron.

LELCs are composed of poorly differentiated cells arranged in solid nests, tubules and trabeculae with poorly demarcated, infiltrative margins. Inflammatory lymphoid infiltrate is extremely abundant and, differently from medullary carcinoma, is intratumoral rather than peritumoral, permeating between neoplastic cells (Figure 3A) [41]. Lymphoid follicles with germinal centers are usually present.

### 6.5. Molecular Genotype

While nasopharyngeal and gastric LELCs are frequently associated with EBV, only in 3/9 colonic cases has this association been reported [42]. Of interest, 2 cases were associated with ulcerative colitis [43] and a further 2 colonic LELCs showed microsatellite instability (1 due to epigenetic methylation of MLH1 promoter and 1 Lynch Syndrome associated) [41,44].

### 6.6. Prognosis

Prognosis of LELCs has been suggested to be more favorable than conventional CRC, but data are few; the role of inflammation, EBV and MSI status requires further investigation [40].

## 7. Cribriform Comedo-Type Carcinoma (CC-Type)

### 7.1. Background

According to the largest reported series by Lino-Silva et al. of 18 cases [45], cribriform comedo-type (CC-type) CRC represents 7.3% of colonic adenocarcinomas. 

### 7.2. Clinical Presentation

Mean age at diagnosis is similar to that of conventional CRC with a predominance in the male sex.

### 7.3. Sidedness

CC-type can be found all along the large bowel with no reported site predilection.

### 7.4. Morphologic Diagnostic Criteria

CC-type CRC is defined as a tumor having “extensive large cribriform glands with central necrosis analogous to breast adenocarcinoma” [18] with tightly packed neoplastic glands, minimal intervening stroma and cribriform architecture. Both histologic hallmarks (cribriform and comedo) are necessary for the CC-type diagnosis (Figure 3B). Its immunoprofile is similar to that of conventional adenocarcinoma (CK20+, CDX2+, MUC2+) [45]. CC-type CRC may be found in combination with micropapillary pattern [46].

### 7.5. Molecular Genotype

The molecular genotype of CC-type CRC has not been specifically investigated however it is usually microsatellite stable with a CIMP profile [47].

### 7.6. Prognosis

CC-type CRC is mainly diagnosed at advanced stage (III, IV) and often shows lymphovascular invasion and nodal metastases. Patients with CC-type CRC have shorter overall survival compared to patients with conventional CRC [45]. Furthermore, the presence of CC-type features in pT1 adenocarcinomas is predictive of nodal metastases [48]. 

## 8. Micropapillary Adenocarcinoma (MPA)

### 8.1. Background

Micropapillary adenocarcinoma (MPA) was firstly reported in 2005 [49] and its incidence varies from 9% to 19% of CRC [50]. Pure micropapillary carcinoma is rare, while a micropapillary component ranging between 5 and 30% in an otherwise conventional adenocarcinoma is the most frequent occurrence [51]. 

### 8.2. Clinical Presentation

MPA is more frequent between 53 and 72 years [52,53], while it is rare in young patients [54].

### 8.3. Sidedness

This variant occurs more frequently in the rectum and the right colon [50,55].

### 8.4. Morphology

At histopathology, MPA is defined by the presence of small, tight, round to oval, cohesive clusters of neoplastic cells floating in clear spaces, lined by delicate strands of fibro-collagenous stroma without endothelial lining and with no evidence of inflammatory cells (Figure 3C). This aspect is probably due to peritumoral tissue retraction and to the reversed polarity of the tumor cell in the clusters (“inside-out” growth pattern) [50,56]. This latter aspect is confirmed by their inverted immunohistochemical MUC1 expression, lack of MUC2 stain and loss or altered pattern of E-cadherin stain. Interestingly, tumour cells express mesenchymal markers (vimentin) and nuclear localization of SMAD4, which suggests epithelial-mesenchymal-transition [56,57,58]. At electron microscopy, neoplastic cells show microvilli on their outer surface, with secretory activity toward the surrounding stroma [52]. Micropapillary features are often maintained in the nodal metastases. Poorly differentiated clusters of tumor cells (PDC) [59], recently described at the periphery or within the tumor mass, display a morphologic similarity to the micropapillary carcinoma, and according to some authors [57], the micropapillary pattern and poorly differentiated clusters may represent the same biological phenomenon. 

### 8.5. Molecular Genotype

MPA shows frequent TP53, KRAS and BRAF-*V600E* mutations, and it develops via classical chromosomal instability (CIN pattern), while MSI is infrequent [52,58].

### 8.6. Prognosis

MPA is characterized by unfavourable prognosis, being frequently diagnosed at advanced stage with high occurrence of lymph node or distant metastases [49,51,56].

## 9. Low Grade Tubuloglandular Adenocarcinoma (LGTGA)

### 9.1. Background

Low-grade tubuloglandular adenocarcinomas (LGTGA) represent approximately 10% of inflammatory bowel disease (IBD) associated CRCs and less than 1% of all CRCs [60].

### 9.2. Clinical Presentation

A slightly higher prevalence in men (1.4:1) has been reported and these tumors are generally diagnosed in middle-aged or older adults (median age 41.5 years, range 28–58) [60]. In some cases, tumors may only be identifiable on random sections of the resected specimen without macroscopic evidence of a cancerous growth. One third of described LGTGA coexisted with one or more synchronous adenocarcinomas of conventional histologic types. LGTGA are more frequently observed in ulcerative colitis than Crohn’s disease and the median duration of IBD before surgery is usually more than 20 years.

### 9.3. Sidedness

Distributed roughly equally on either side of the splenic flexure, LGTGA has been described to occur also in the terminal ileum [61].

### 9.4. Morphologic Diagnostic Criteria

LGTGAs are characterized by very well-differentiated invasive glands with uniform circular or tubular profiles and they are usually surrounded by flat or polypoid low-grade dysplasia. The neoplastic epithelia show bland cytologic atypia resembling overlying low-grade or indefinite dysplastic crypts and the tumor is associated with little to no stromal desmoplasia (Figure 3D). Heterogeneous tumors containing well-defined regions of LGTGA and adjacent conventional carcinoma are also seen, with the conventional component typically located in deeper regions of the tumor, suggesting histologic progression from a lower to a higher grade of neoplasia. Immunohistochemically LGTGA present frequent co-expression of CK7 and CK20 (69%).

### 9.5. Molecular Genotype

IDH1 mutations were observed in three LGTGA of a series of eight cases, and in two of these three cases a concomitant activating KRAS mutation was also present [62]. High prevalence of loss of MLH1 expression by immunohistochemistry (55%) has been reported supporting an association with MSI-H status [60].

### 9.6. Prognosis

Limited information is available; generally a favorable prognosis has been described unless a synchronous conventional colorectal carcinoma is also present.

## 10. Villous Carcinoma (VC)

### 10.1. Background

Villous carcinoma (VC) is a rare (1%), well differentiated subtype of CRC, resembling villous adenoma on the surface and it is also known as “adenoma-like adenocarcinoma” or “‘papillary adenocarcinoma” [63,64,65].

### 10.2. Clinical Presentation

VC shows male predominance; the median age at diagnosis is 66 years (range 48 to 83 years), which is comparable to conventional CRC [63].

### 10.3. Sidedness

Contrasting data is present in the literature; a study has shown a predilection for the rectum and sigma (almost the 80% of the cases).

### 10.4. Morphologic Diagnostic Criteria

**Figure 4:**
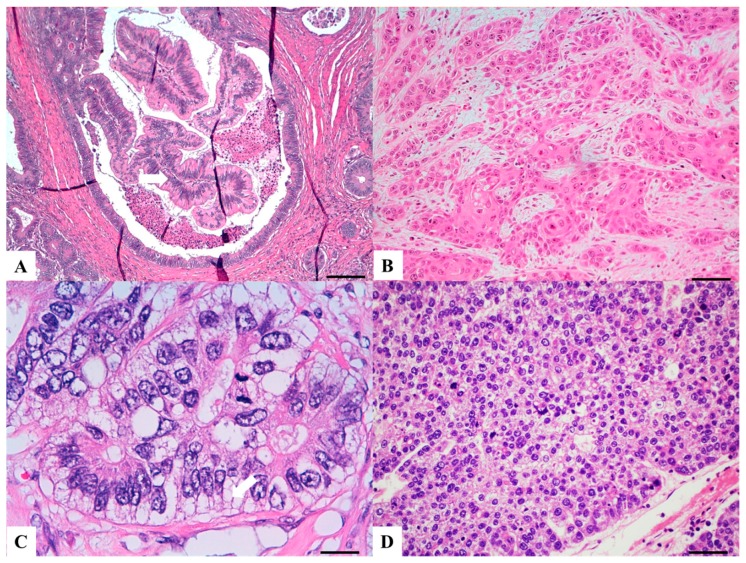
Haematoxylin and Eosin stained sections of rare type colorectal carcinomas. (**A**) Villous carcinoma: invasive carcinoma with villous features consisting of usually intraglandular papillary projections (yellow arrow) associated with an expansile growth pattern, at the deep portions of the tumor. Scale bar 400 micron. (**B**) Squamous carcinoma: morphologically similar to other squamous cell carcinomas occurring in other organs with possible keratinization. Scale bar 200 micron. (**C**) Clear cell carcinoma: clear cell cytoplasm identified in polygonal cells with a central nucleus, columnar cells with an eccentric nucleus (red arrow) and/or round/oval cells with abundant cytoplasm and inconspicuous marginally located nucleus similar to lipocytes or lipoblasts. Scale bar 50 micron. (**D**) Hepatoid carcinoma: large polygonal-shaped cells, with granular eosinophilic cytoplasm, prominent nucleoli and trabecular and pseudo-acinar growth pattern similar to hepatocarcinoma. Scale bar 200 micron.

VC is an invasive carcinoma with architectural and cytologic features resembling villous adenoma. The neoplastic epithelium is more frequently characterized by low-grade atypia and intraglandular papillary projections associated with an expansile growth pattern are usually seen (Figure 4A). The presence of neoplastic epithelial islands surrounded by desmoplastic stroma may help in the diagnosis [63] which may be challenging in biopsy specimens. The lesions often have a pushing border involving the wall of the colon. Most lesions have non-adenoma-like areas, which can display dilated glands filled with mucin at the leading edge of the tumor or may be represented by well-differentiated CRC of no particular subtype [66].

### 10.5. Molecular Genotype

In a series of 24 cases, KRAS mutations were detected in 14 (58%) cases and MSI was present in 4 of 17 (24%) [66].

### 10.6. Prognosis

VC shows favorable prognosis with a lower rate of nodal and distant metastases compared to conventional CRC [63].

## 11. Squamous (SCC) and Adenosquamous Carcinoma (ASC)

### 11.1. Background

Both lesions are rare, representing 0.1–0.5% of primary CRC [67,68]. For squamous cell carcinoma (SCC), diagnosis requires the exclusion of any involvement of cloacogenic or anal squamous carcinoma, the absence of any squamous cell carcinoma elsewhere and thorough extensive sampling of the lesion to exclude adenosquamous carcinoma (ASC).

### 11.2. Clinical Presentation

SCC is associated with ulcerative colitis, chronic colo-cutaneous fistula, schistosomiasis and colonic duplication [67]; ASC can occur in patients with ulcerative colitis [68] and both may cause paraneoplastic hypercalcemia [69,70]. There is no significant difference in sex, age, and ethnicity between SCC/ASC and conventional CRC.

### 11.3. Sidedness

ASC typically arises in the right colon, whereas SCC is most commonly located in the proximal colon and rectum.

### 11.4. Morphologic Diagnostic Criteria

SCC is morphologically similar to squamous cell carcinomas occurring in other organs and basaloid or acantholytic subtypes have been described (Figure 4B). ASC, on the other hand, resembles conventional colorectal adenocarcinoma, but with areas of squamous differentiation, either admixed or distinct. Both histotypes are positive for CK5/6 and p63 in the squamous component [71].

### 11.5. Molecular Genotype

HPV infection does not appear to play a role. No information is available on the molecular landscape of these histotypes [72,73].

### 11.6. Prognosis

Both SCC and ASC have higher metastatic rates and worse prognosis than conventional CRC and tumor stage is often advanced at onset [67,68].

## 12. Primary Clear Cell Carcinoma (CCC)

### 12.1. Background

Primary colorectal clear cell carcinoma (CCC) is a rare CRC type with 37 cases described in the Literature since 1944 [74]. Two tumor types of CCC can be recognized on the basis of different immunoprofiles: (a) intestinal CCC (iCCC) and (b) Müllerian CCC (mCCC) [74].

### 12.2. Clinical Presentation

The mean age of onset is 56.5 years (range of 27–89); in particular women are affected at a younger age than males (51.9 years versus 61.5 years) [74].

### 12.3. Sidedness

All colorectal sites may be involved however the left side is more frequent. mCCCs arise exclusively in the rectum (77%) or sigma (23%) of women [74].

### 12.4. Morphologic Diagnostic Criteria

The diagnostic hallmark of CCC is the presence of more than 50% of clear cells (Figure 4C) as polygonal cells with a central nucleus, columnar cells with an eccentric nucleus and/or round/oval cells with abundant cytoplasm and inconspicuous marginally located nucleus similar to lipocytes or lipoblasts. Both a “pure form” and a “composite” form, admixed with a conventional CRC, have been described. iCCC diagnostic criteria are: (a) composite CCC with conventional adenocarcinoma or presence of adenomatous component [75], (b) absence of adjacent endometriosis, (c) intestinal immunoprofile (CK20+, CK7−, CEA+, CDX-2+) [74,76]. mCCC diagnostic criteria are: (a) evidence of Müllerian origin immunoprofile (CK7+, CK20−, CEA−, CA125+) with or without histologically recognizable endometriotic foci in close proximity; (b) exclusion of origin from other primary sites [74,77]. 

### 12.5. Molecular Genotype

iCCC show predominantly (80%) KRAS mutations and proficient MMR profile. mCCC have not been studied as yet.

### 12.6. Prognosis

While iCCC shows aggressive behavior (mean overall survival of 13.8 months): 36% are diagnosed with locally advanced disease and 33% show distant metastases at onset. mCCC, on the other hand, is thought to be an indolent tumor [74].

## 13. Hepatoid Adenocarcinoma (HepAC)

### 13.1. Background

HepAC is a rare extrahepatic adenocarcinoma mimicking hepatocellular carcinoma (HCC), first described in 1970 [78]. In the gastrointestinal tract, HepAC is more frequent in the stomach [79], possibly due to a common foregut embryologic origin, while only 42 cases affecting the bowel have been described.

### 13.2. Clinical Presentation

A comprehensive review [80] has shown that intestinal HepAC commonly occurs in younger patients (around 50 years of age) with a male predominance. Very high serum Alpha Fetoprotein (AFP) levels are reported in almost all cases. Furthermore, an association with long standing inflammatory bowel disease has been reported (8/42 patients), suggesting its possible role in HepAC cancerogenesis.

### 13.3. Sidedness

About 70% of reported cases arise in the colon (50%) or rectum (20%) while 30% of cases involve the small bowel. 

### 13.4. Morphologic Diagnostic Criteria

HepAC is characterized by large polygonal-shaped cells, with granular eosinophilic cytoplasm, prominent nucleoli and trabecular and pseudo-acinar growth patterns (Figure 4D). Morphology resembles HCC and this can cause diagnostic pitfalls, especially when presenting as liver metastases. Immunohistochemistry is mandatory to reach a correct diagnosis with AFP, glypican-3, cytokeratins 18, 19 and carcinoembryonic antigen (CEA) positivity in nearly all cases [80,81] while Hep Par1 positivity is reported only in about 40% of cases. Little information is available on intestinal markers, with frequent CK20 negativity; CDX-2 positivity is reported only in a few cases (the majority of cases were not tested).

### 13.5. Molecular Genotype

Little in know about the molecular profile and pathogenesis of colorectal HepAC is still an enigma. Possible hypotheses include derivation from dispersed fetal cells within the tumor or the activation of silenced liver specific genes during carcinogenesis [82].

### 13.6. Prognosis

HepAC is aggressive with markedly worse prognosis compared to conventional CRC. The majority of patients (80%) present in stages III or IV and distant metastases (mainly hepatic) at onset are reported in 40% of patients. Patients generally relapse and succumb within the first year. Radical surgery with liver resection of metastatic disease, followed by chemotherapy, is considered the first choice treatment though HepAC seems to show no response to conventional CRC chemotherapy [80,81,82].

## 14. Primary Colorectal Choriocarcinoma (pChC)

### 14.1. Background

Primary choriocarcinoma is a highly malignant neoplasm with trophoblastic differentiation which typically occurs either associated with pregnancy or as ovarian/testicular germ cell tumors. Extra-gestational, non-gonadal primary choriocarcinomas are exceedingly rare and may be found in different sites such as the mediastinum, retroperitoneum or lung; in the digestive system, the stomach is the most frequent site while only 29 primary colorectal choriocarcinomas (pChC) have been described in the literature.

### 14.2. Clinical Presentation

Patients are usually younger than conventional CRC (median 54 years, range 12–74; 45% are <50 years of age) with no gender predilection. Symptoms are similar to conventional CRC however most patients have increased serum βHCG levels (which may be used as a biomarker) [83]. Three cases have been found to be associated with Crohn’s disease or ulcerative colitis.

### 14.3. Sidedness

pChC are equally distributed along the length of the colon-rectum (34% in the right colon; 41% in the left colon; 24% in the rectum).

### 14.4. Morphologic Diagnostic Criteria

**Figure 5:**
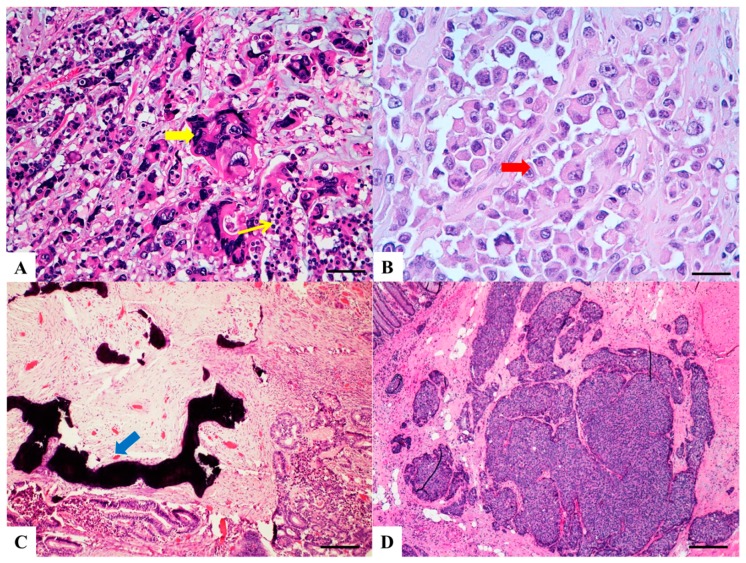
Haematoxylin and Eosin stained sections of rare type colorectal carcinomas. (**A**) Colorectal Choriocarcinoma: biphasic solid nests and trabeculae of mononucleated cells with clear cytoplasm (thin yellow arrow) and pleomorphic cells with abundant vacuolated or eosinophilic cytoplasm and single or multiple vescicular nuclei with conspicuous nucleoli (thick yellow arrow). Scale bar 200 micron. (**B**) Rhabdoid Colorectal Carcinoma: rhabdoid cells characterized by a large, eccentrically located nuclei, prominent nucleoli (red arrow) and abundant eosinophilic cytoplasm. Scale bar 100 micron. (**C**) Carcinoma with osseous metaplasia: osseous metaplasia (blue arrow) is recognized in conventional CRC as foci of bone formation in the stroma, with calcification, osteoid matrix, osteoclasts and osteoblasts. Scale bar 400 micron. (**D**) Undifferentiated carcinoma: sheets of undifferentiated cells showing a variable grade of pleomorphism with no gland formation, mucin production or other line of differentiation. Scale bar 400 micron.

pChC are usually large, solid masses with necrosis and haemorrhages. At histology, these tumors present as either: mixed adenocarcinomatous and choriocarcinomatous components (72% of cases) or show pure choriocarcinomatous features (28% of cases). The defining βHCG immunopositive component is characterized by biphasic solid nests and trabeculae of mononucleated cells with clear cytoplasm and pleomorphic cells with abundant vacuolated or eosinophilic cytoplasm and single or multiple vescicular nuclei with conspicuous nucleoli (Figure 5A). Diffuse vascular invasion, extensive necrosis and numerous typical/atypical mitoses are also seen.

### 14.5. Molecular Genotype

Three theories of development have been proposed [84,85]: (1) primordial germinal cells migrated to anomalous sites during embryogenesis; (2) primary unknown gestational or gonadal lesions; (3) the most accredited theory is dedifferentiation of a preexisting colonic adenocarcinoma [86]. In consideration of the few cases reported no information is available on specific molecular findings.

### 14.6. Prognosis

Disease progression is generally rapid with early metastatic dissemination (often at diagnosis): distant metastases predominantly show pure choriocarcinomatous features. Chemotherapeutic regimens targeting either colorectal germ cell tumors have been proposed with variable results, nonetheless survival is less than 1 year from onset [87]. 

## 15. Rhabdoid Colorectal Carcinoma (RhC)

### 15.1. Background

Rhabdoid colorectal carcinoma (RhC) is a rare and lethal neoplasm morphologically similar to malignant extrarenal rhabdoid tumor. Thirty-four cases of colorectal RhC have up till now been described [88].

### 15.2. Clinical Presentation

RhC occurs in adulthood (31–87 years) around the 7^th^ decade without gender predilection.

### 15.3. Sidedness

These tumors predominantly affect the right colon [89].

### 15.4. Morphologic Diagnostic Criteria

The diagnostic hallmark of this neoplasm is the presence of “rhabdoid” cells characterized by a large, eccentrically located nuclei, prominent nucleoli [88], abundant eosinophilic cytoplasm which, at ultrastructural analysis shows aggregates of intermediate filaments (Figure 5B) [90]. The extent and distribution of the rhabdoid component is highly variable, ranging from “composite carcinomas,” in which the rhabdoid elements are associated with adenocarcinoma, to “pure” rhabdoid carcinomas without an evident conventional component [88]. A quota of RhCs show loss of immunoexpression of INI1; however this may also be seen in a small group (11%) of conventional CRC [91].

### 15.5. Molecular Genotype

RhCs show predominant BRAF mutations and MSI-H molecular profile. In this specific genotypic asset, alterations in chromatin remodeling (SWI/SNF or SMARCB1) complex [91,92] and in the centrosome structure (Ciliary Rootlet Coiled Coil; CROCC) have been reported as major genetic determinants of rhabdoid pathogenesis [89]. 

### 15.6. Prognosis

RhC prognosis is very aggressive with an overall survival of 7.9 months regardless of stage [89]. The presence of lamellipodia in CROCC-mutated RhC may explain their aggressive behavior [90].

## 16. Carcinoma with Osseous Metaplasia (COM)

### 16.1. Background

Primary CRC with presence of heterotopic bone formation, initially described by Hasegawa [93] in 1923 and subsequently reported by Dukes [94] in 1939, is a rare event with a suggested incidence of 0.4% in rectal cancers. From 1990 to 2019, 21 cases have been described and the largest published series [95] is composed of just three cases, with incidence hypothesized to be as low as 0.15%.

### 16.2. Clinical Presentation

No gender predilection has been reported and age at onset is variable (29 to 90 years). Symptoms and endoscopy are no different to conventional CRC, while calcification/bone formation may be seen at imaging [96].

### 16.3. Sidedness

COM is more frequently diagnosed in the rectum (about 40% of cases), followed by left and right colon and appendix.

### 16.4. Morphologic Diagnostic Criteria

Osseous metaplasia, recognized as foci of bone formation in the stroma, with calcification, osteoid matrix, osteoclasts and osteoblasts, has been reported in conventional CRC or, less frequently, in other colorectal histotypes, such as serrated carcinoma (Figure 5C). A possible intriguing explanation of osseous metaplasia is that undifferentiated stromal cells transform into osteoblasts under the stimulus of bone morphogenetic proteins (BMP 5 and 6) produced by cancer cells [97].

### 16.5. Molecular Genotype

Mutations in different codons of KRAS have been detected, however data are too few to suggest a role of KRAS mutations in COM.

### 16.6. Prognosis

Data on prognosis are scarce, however osseous metaplasia does not seem to affect prognosis [96].

## 17. Spindle Cell Carcinoma (SpCC)

### 17.1. Background

Spindle cell carcinoma (SpCC) (alternatively named mesenchymal or sarcomatoid carcinoma) is a rare variant of CRC, with less than 20 cases reported up to now [98,99,100].

### 17.2. Clinical Presentation

SpCC has mean age at presentation similar to that of conventional CRC with no gender predilection [98,99,100].

### 17.3. Sidedness

SpCC shows a slight predominance in the left colon and rectum [98]. 

### 17.4. Morphologic Diagnostic Criteria

SpCC is defined as a “biphasic carcinoma with a spindle-cell sarcomatoid component in which the tumor cells are at least focally immunoreactive for keratins” [18]. Although the name carcinosarcoma has been used interchangeably [99], it should be reserved to tumors with absence of epithelial differentiation (absence of epithelial markers) in the sarcomatous component [98]. The rarity of SpCC may be due to its histological similarity to mesenchymal tumors which renders its differential diagnosis quite challenging without immunohistochemistry.

### 17.5. Molecular Genotype

The molecular features of this variant have not been specifically investigated. 

### 17.6. Prognosis

SpCC is associated with low overall survival, with most patients surviving less than 24 months [98,99,100].

## 18. Pleomorphic Carcinoma (PlC)

### 18.1. Background

Pleomorphic adenocarcinoma (PlC) was firstly described in the colon in 1989 [101] with the report of two cases, and thereafter only a further two cases have been reported [102,103]. 

### 18.2. Clinical Presentation

Age of presentation ranges between 67 and 86 years; three patients with pleomorphic CRC were females and one was a male [101,102,103].

### 18.3. Sidedness

Two cases involved the caecum, one was diagnosed in the descending colon and one in the sigmoid colon [101,102,103]. 

### 18.4. Morphologic Diagnostic Criteria 

Histologically, colonic PlC shows pleomorphic gemistocytic giant cells and spindle cells and it may resemble choriocarcinoma and sarcoma. Positive staining for cytokeratins and absence of staining for βHCG are helpful to confirm the diagnosis. PlC is not currently listed among CRC variants in the WHO classification and whether this is a separate entity or whether it may be included together with spindle cell/mesenchymal carcinomas is unknown but likely. 

### 18.5. Molecular Genotype

Molecular features of this variant have been not investigated.

### 18.6. Prognosis

Although two patients with pleomorphic carcinoma were reported to have short survival [101,102], no definitive conclusions on the prognostic value of this variant can be drawn due to its rarity. 

## 19. Undifferentiated Carcinoma (UC)

### 19.1. Background

Undifferentiated carcinomas (UCs) are colorectal tumors showing no gland formation, but still presenting features of epithelial differentiation [18]. Some Authors include in this category tumors showing minimal (generally less than 5%) glandular architecture.

### 19.2. Clinical Presentation

Patients’ age is similar to conventional CRCs; no gender predilection has been reported.

### 19.3. Sidedness

UCs occur throughout the colon-rectum but they are more frequently detected in the right colon [104].

### 19.4. Morphologic Diagnostic Criteria

UCs consist of sheets of undifferentiated cells showing a variable grade of pleomorphism with minimal gland formation, mucin production or other lines of differentiation (e.g., squamous etc.) and for this reason extensive sampling is required (Figure 5D). Sometimes they display a trabecular pattern, they are often characterized by an infiltrative pattern of growth and show extensive necrosis. Positive staining for cytokeratins is required to confirm the diagnosis. Pure UCs are very rare, but adenocarcinomas containing an undifferentiated component are encountered more often. UCs should be distinguished from medullary carcinomas.

### 19.5. Molecular Genotype 

Little evidence is present with regards to molecular features.

### 19.6. Prognosis

UCs are aggressive tumors, showing a low 5-year cancer specific survival [104]. In the setting of metastatic disease patients with UCs also have a worse prognosis [105].

## 20. Conclusions

A better recognition and histological characterization of CRC is becoming mandatory as new molecular pathways which identify tumors with different prognoses and treatment are emerging. Pathologists are being asked to separate variant CRCs from conventional type CRCs indicating that, even though we are becoming ever more molecular minded, morphology still plays an important role. We seem to be “going back to the future”: the more we take the molecular path ahead the more we seem to rely on the morphological lights which guide our way.

## Figures and Tables

**Table 1:** Clinico-pathologic and immune-molecular characteristics of rare type colorectal carcinomas.

Histotype		Mean Age	Site	Prognosis^#^	Main Diagnostic Criteria	Immunoprofile	Molecular Profile
**Serrated Adenocarcinoma (SA)**		67	Right colon and rectum	Aggressive	Epithelial serrations +/− tufts; eosinophilic cytoplasm; vescicular nuclei		KRAS^mut^ BRAF^mut^ MSI
**Mucinous (colloid) adenocarcinoma** (MA)		60	Right colon	Similar to conventional	Abundant extracellular mucin in more than 50%	MMRd, PDL1+	KRAS^mut^ BRAF^mut^, MSI
**Signet ring cell carcinoma** (SRC)		65	No site predilection	Aggressive	Signet ring cells in more than 50%	MMRd, PDL1+	KRAS^mut^ BRAF^mut^, MSI
**Medullary carcinoma** (MC)		70	Right colon	Favourable	Solid growth pattern with circumscribed tumor borders; tumor cells with a syncytial appearance; conspicuous intra and peri tumor lymphocytes.	MMRd, CDX-2−, CK20+, calretinin+	MSI, BRAF^mut^
**Lymphoepitelioma-like** (LELC)		62	No site predilection	Favourable	Poorly differentiated carcinoma with abundant intratumour infiltrating lymphocytes; presence of EBV	MMRd	EBV+
**Cribriform comedo-type adenocarcinoma** (CC-type)		56	No site predilection	Aggressive	Tightly packed neoplastic glands and cribriform architecture and large glands with central necrosis	CK20+, CDX-2+, MUC2+	
**Micropapillary carcinoma** (MPA)		69	Right colon and rectum	Aggressive	Clusters with lacunar space of more than 5 neoplastic cells; inverse polarity	Inverted MUC1, MUC2−, E-cadherin altered pattern	TP53^mut^, KRAS^mut^, BRAF^mut^, CIN
**Low -grade tubuloglandular carcinoma** (LGTGA)		42	No site predilection	Favourable	Tubular architecture composed of neoplastic glands with little atypia	MMRd (MLH1d)	MSI, KRAS^mut^, IDH1^mut^
**Villous carcinoma** (VC)		66	Left colon	Favourable	Villous architecture in >50%		KRAS^mut^
**Squamous/Adenosquamous carcinoma** (SCC/ASC)		60	Right colon	Aggressive	Squamous differentiation either pure or composite with glandular component	p63+, CK5/6+	
**Clear cell carcinoma**	**Mullerian-**mCCC	52	Exclusively rectum	Favourable	Clear cells in more than 50%; endometriosis or pregnancy	CK20−, CK7+, CEA−, CA125+	
	**Intestinal-**iCCC	61	No site predilection	Aggressive	Clear cell in more than 50%	CK20+, CK7−, CEA+, CDX-2+)	KRAS^mut^, MSI
**Hepatoid carcinoma** (HepAC)		50	Rectum	Aggressive	Neoplastic cells with hepatoid appearence in solid, trabecular o pseudoacinar architectural patterns	AFP+ (also serum), Glypican-3+, CK18+, CK19+, CEA+, Hep Par1 + (40%)	
**Primary Choriocarcinoma** (pChC)		54	Left colon	Aggressive	Syncytiotrophoblast-like cells	β-HCG (also serum)	
**Rhabdoid carcinoma** (RhC)		70	Right colon	Aggressive	Rhabdoid cells >5%	CK20−, Vimentin+, CDX-2−, INI1−, CROCC reduction signals.	BRAF^mut^, MSI, CROCC^mut^
**Carcinoma with osseous metaplasia** (COM)		58	Left colon	Similar to conventional	Presence of osseous metaplasia in a conventional adenocarcinoma		
Spindle cell or mesenchymal carcinoma* (SpCC)		70	Left colon and Rectum	Aggressive	Biphasic carcinoma with a spindle-cell sarcomatoid component (cytokeratin +); may have giant cells	Vimentin+, CK+ (focal)	
**Undifferentiated carcinoma** (UC)		70	No site predilection	Aggressive	Evidence of epithelial differentiation with minimal or without gland formation	CK+, absence of other differentiation markers	

* Pleomorphic carcinoma is considered within this type. # Prognosis is compared to conventional colorectal adenocarcinoma. MSI—microsatellite instability; MMRd—mismatch repair protein deficiency; CK—cytokeratins; EBV—Ebstein-Barr virus; CIN—Chromosomal instability.
